# World Checklist of Opiliones species (Arachnida). Part 2: Laniatores – Samooidea, Zalmoxoidea and Grassatores *incertae sedis*

**DOI:** 10.3897/BDJ.3.e6482

**Published:** 2015-12-21

**Authors:** Adriano B. Kury, Daniele R. Souza, Abel Pérez-González

**Affiliations:** ‡ Museu Nacional, Universidade Federal do Rio de Janeiro, Rio de Janeiro, Brazil; § MACN - Museo Argentino de Ciencias Naturales "Bernardino Rivadavia", Buenos Aires, Argentina

**Keywords:** Neotropics, Indo-Malaya, Afrotropics

## Abstract

Including more than 6500 species, Opiliones is the third most diverse order of Arachnida, after the megadiverse Acari and Araneae. This database is part 2 of 12 of a project containing an intended worldwide checklist of species and subspecies of Opiliones, and it includes the members of the suborder Laniatores, infraorder Grassatores of the superfamilies Samooidea and Zalmoxoidea plus the genera currently not allocated to any family (i.e. Grassatores *incertae sedis*). In this Part 2, a total of 556 species and subspecies are listed.

## Introduction

This work is a presentation to the 2nd part of the database of the valid species of harvestmen in the World. Two important superfamilies of Grassatores are listed here, along with all species of the infraorder that could not be allocated to any of the known families. Mandatory taxonomic chages are made in a sister paper specifically dedicated to the formalization and documentation of these nomenclatural acts (Kury and Pérez-González 2015. The suborder Laniatores is composed of the so-called "spiny" or "armored" harvestmen, although many members of suborders Eupnoi or Dyspnoi also match this characterization. They are usually divided into the two infraorders, Insidiatores and Grassatores, but the former is mostly considered non-monophyletic (Kury 2015, Sharma and Giribet 2011). The deeper relationships among Grassatores are still fluctuating, although some superfamilies can be recognized with a reasonable degree of accuracy, as in Sharma and Giribet (2011): Gonyleptoidea (BS = 57%, PP = 1.00), Assamioidea (PP = 0.95). Phalangodidae and Sandokanidae are basal isolated families, while the SE Asian families are more or less loosely grouped in Epedanoidea (PP = 0.77). A consensus is illustrated as figure 1 in Sharma and Giribet (2014)

### Placement and composition

The history of the families which now compose the Samooidea and Zalmoxoidea is rather complex, with many generic transfers between them. Older authors such as Sørensen and Thorell described isolated families mainly from SE Asia. Roewer (1912) considered all of them to be subfamilies of an immense and meaningless Phalangodidae or even merged some in Phalangodinae. In an unpublished dissertation Kury (1993) recognized a large superfamily Zalmoxoidea, including Biantidae, Minuidae, Podoctidae, Samoidae, Stygnommatidae and Zalmoxidae, but this suprafamilial name only appeared in press much later (Kury and Cokendolpher 2000). In Kury’s catalogue (Kury 2003), the families of Grassatores were not included in superfamilies. Only in a synoptic work by Giribet and Kury (2007), were the superfamilies of Grassatores explicitly defined for the first time. They recognized Zalmoxoidea as (Icaleptidae, Guasiniidae, Zalmoxidae, Fissiphalliidae), while the Samooidea included (Samoidae, Podoctidae, Biantidae, Minuidae, Stygnommatidae). There was a lapse with the name of this latter superfamily being called either Samooidea or Biantoidea in the same book. This happened because composition was uncertain, and in the last minute Samoidae was also included, being older than Biantidae. Giribet et al. (2010) recovered a paraphyletic Samooidea (Biantidae, Escadabiidae, Kimulidae, Samoidae, Stygnommatidae) respective to the Zalmoxoidea, while they included in Zalmoxoidea the same Fissiphalliidae, Guasiniidae, Icaleptidae, and Zalmoxidae, with a paraphyletic Samooidea as sister group. In that paper they also found that Podoctidae should be placed far from both, isolated among the basal Grassatores. Later, Sharma and Giribet (2011) recovered a reduced monophyletic Samooidea with only 3 families (Biantidae, Samoidae and Stygnommatidae) while they augmented Zalmoxoidea transfering Escadabiidae and Kimulidae to it, resulting in 6 included families. Recent molecular analyses recovered a sister-group relationship between Samooidea and Zalmoxoidea (*e.g.*, Sharma and Giribet 2011, with BS = 85% and PP = 1.00). This hypothesis is also supported by morphology (Pérez-González and Kury 2007b). However, the support for each superfamily is unequal. On one hand we have evidence for a strong Zalmoxoidea *sensu* Sharma and Giribet (Sharma and Giribet 2011) (BS = 89%, PP = 1.00) whereas Samooidea it is recovered as monophyletic but with limited support (BS = 53%, PP = 0.62) (Sharma and Giribet 2011). That could be a reflection that whereas the taxon sampling for Zalmoxoidea has been greatly enhanced lately (Sharma and Giribet 2011, Sharma and Giribet 2012) the taxon sampling for Samooidea still remains unsatisfactory. More effort is needed in studying and sequencing African and Indo-Pacific lineages (Sharma and Giribet 2011), as well as enhancing the representation of Neotropical and Australasian terminals in order to test Samooidea as a natural group and improve the hypotheses of relationship inside the Samooidea/Zalmoxoidea clade.

### Superfamily Zalmoxoidea

**Escadabiidae.** This small Brazilian family was created by Kury & Pérez (in Kury 2003) for 4 genera of Phalangodinae from northeastern Brazil. Kury and Pérez-González (2007) also included *Spaeleoleptes* H. Soares, 1966, which was originally in Phalangodidae Minuinae and latter in *incertae sedis* as per Kury (2003). Kury et al. (2010) further included the monotypic *Brotasus* Roewer, 1928, then in Grassatores *incertae sedis*, but originally in Phalangodidae, Phalangodinae. Escadabiidae was placed in Samooidea by Giribet and Kury (2007), Kury and Pérez-González (2007) and Giribet et al. (2010), but finally it was moved to Zalmoxoidea (Sharma and Giribet 2011), a transfer which has been subsequently supported (Sharma and Giribet 2014), although monophyly remains uncertain. Sharma and Giribet (2012) did not recover *Baculigerus* H. Soares, 1979 as monophyletic.

**Fissiphalliidae.** Martens (1988) erected this micro-diverse family for three new species placed into the single genus *Fissiphallius*, all from the vicinity of Bogotá, Colombia. He discussed other families in Grassatores, but allied Fissiphalliidae most closely with the Podoctidae (a group now considered to be phylogenetically distant from both Zalmoxoidea and Samooidea). The almost simultaneous publication of the resurrection of the Zalmoxidae (Staręga 1989) must have prevented any comparison between each other. Kury and Pérez-González (2002) included Fissiphalliidae in a clade along with Zalmoxidae and Icaleptidae, and commented that keeping Fissiphalliidae as a distinct family could render Zalmoxidae paraphyletic. Giribet and Kury (2007) formally included Fissiphalliidae in Zalmoxoidea closest to Zalmoxidae, and molecular analyses (*e.g.* Sharma and Giribet 2011, 2015) corroborated this view. In the last decade four other species have been described from Brazil, and placed in the same original genus, but without any groundbreaking phylogenetic discussion.

**Guasiniidae.** González-Sponga (1997) proposed this new micro-diverse family for two Venezuelan species, with one genus from Isla Guasina at sea level, another from a tepui (tepuis are table-top mountains found in the Guiana Highlands of South America, especially in Venezuela and western Guyana), 1350 m high. He compared it chiefly with Oncopodidae, without proposing any serious phylogenetic discussion, although the Oncopodidae (now Sandokanidae) are currently considered as an evolutionarily distant and archaic lineage. Pinto-da-Rocha and Kury (2003) added a third species from Brazilian Amazonia, and related Guasiniidae with the Zalmoxidae, Fissiphalliidae and Icaleptidae, a clade established shortly before (Kury and Pérez-González 2002). Giribet and Kury (2007) formalized its inclusion in Zalmoxoidea, a placement subsequently supported by molecular and mophological studies.

**Icaleptidae.** Another micro-diverse family, with only two described Andean species in two genera. In the original description, Kury and Pérez-González (2002) stated: “Among Grassatores, Icaleptidae is most closely related to Zalmoxidae and Fissiphalliidae”. Although they did not cite any superfamilial assignment. Icaleptidae was assigned to Zalmoxoidea very early (Giribet and Kury 2007) and this placement has never been challenged. Icaleptidae was found sister to one unidentified species of the genus *Costabrimma* from Costa Rica, and of uncertain affinities by Giribet et al. (2010). Many unidentified "*Icaleptes"* sp. are nested with two *Costabrimma* in Sharma and Giribet (2012). However, the flea like IV leg used to identify is not anymore a diagnostic character for this family, and it is present in several other Zalmoxoidea taxa, therefore the accurate familial identification of specimens in those later studies needs to be confirmed using genital characters together with other morphological features. *Costabrimma* may represent a lineage of Zalmoxoidea, not yet studied under a revisionary morphological viewpoint. This genus has not been used in the most recent analysis of Sharma and Giribet (2011), where Icaleptidae appeared as sister group to Zalmoxidae + Fissiphalliidae

**Kimulidae.** Sørensen in Henriksen (1932) created this family as Minuidae with 7 new genera, mostly from Venezuela and only one from southern Brazil. Mello-Leitão (Mello-Leitão 1933, Mello-Leitão 1938) subdivided some of Sørensen’s genera and removed *Microminua* and *Minuides* from Minuinae. Roewer never cited Minuinae either as a separate family or as a subfamily of Phalangodidae. H. Soares (1966) expanded the Brazilian representation of Minuinae, describing two new genera from southeastern Brazil. Šilhavý (1978) and González-Sponga (1987) considered both subfamilies Minuinae and Minuidinae as synonyms of Phalangodinae. Kury (1995) started to shrink the Minuidae by removing the original southern Brazilian genus *Phera* to the Gonyleptidae. Kury (2003) reinstated Minuidae as a separate family, expanding it with the inclusion of some Caribbean and Venezuelan genera of Phalangodinae, and both genera of Minuidinae, while removing 3 other genera, including the two remnant Brazilian genera. In 2007 Pérez-González, Kury, and Alonso-Zarazaga (in Pérez-González and Kury 2007a) detected that the type genus was invalid due to homonymy (replaced by *Minuella*) and were forced to change the family name to Kimulidae. Pérez-González and Kury (2007a) newly included *Tegipiolus* from NE Brazil in Kimulidae. They also transferred *Minuides* to the Zalmoxidae, automatically carrying the synonymy of the Minuidinae. *Kimula* has been chosen as type genus, leading to the new name of this small family, now comprised of nine genera.

**Zalmoxidae.** Sørensen (1886) created the new family Zalmoxioidae only for the new genus *Zalmoxis*, with two species from Fiji. Soon afterwards, Thorell (1889) synonymized this with family Epedanoidae and three years later, Loman (1902) included *Zalmoxis* in an expanded Epedanidae with many genera. Roewer (1912) included *Zalmoxis* as a genus of his Phalangodinae within the immense family Phalangodidae. During eight decades, *Zalmoxis* and closely related genera remained buried in Phalangodinae while other poor arrangements were made regarding the small Grassatores, and while a legion of would-be Zalmoxidae were described under Phalangodinae. Mello-Leitão (1933) erected the subfamily Minuidinae in Phalangodidae, but later (Mello-Leitão 1938), he wrote: “Minuidinae (n. subfam.)”. This taxon (Mello-Leitão 1938) was formed by two genera sorted from Sørensen’s Minuidae — *Minuides* and *Pseudominua*. H. Soares (1972) created Stygnoleptinae as a new subfamily of Gonyleptidae containing 3 entirely distant genera – *Glysteroides* Roewer, 1943 (currently in Gonyleptidae), *Saramacia* Roewer, 1913 (currently in Manaosbiidae), and *Stygnoleptes* Banks, 1914 (currently in Zalmoxidae). Staręga (1989) finally resurrected Zalmoxidae to include 5 Paleotropical genera, remarking that a great number of genera had been sunken into *Zalmoxis* by Goodnight and Goodnight (1957). Staręga (1992) included 3 more Afrotropical genera from Seychelles and Madagascar. Kury (1997) dismantled the Stygnoleptinae, synonymizing this name with Zalmoxidae, and proceeded in subsequent studies (Kury and Cokendolpher 2000, Kury 2003) to promote a mass exodus from the Phalangodidae into Zalmoxidae, to expand into what is now the largest family of Zalmoxoidea. Pérez-González and Kury (2007a) presented evidence that *Minuides* is a Zalmoxidae, therefore Minuidinae is a synonym of this family. Sharma and Giribet (2012) demonstrated that the family Zalmoxidae, “similar to the Pacific iguanas, constitutes the unusual case of a lineage of Neotropical origin that colonized the Indo-Pacific, likely by ancient transoceanic dispersal during the Late Cretaceous.” Accordingly, Sharma and collaborators (Sharma et al. 2011a, Sharma 2012, Sharma et al. 2012) while describing many new Paleotropical species, sought to merge all Old World genera of Zalmoxidae to better accommodate this single origin within a wider familial clade,which is much more diverse in the Neotropics.

### Superfamily Samooidea

**Biantidae.** Karsch (1880) described the genus *Hinzuanius* from the Comoros Islands in the family Gonyleptoidae. Pavesi (1884) described a second species from Ethiopia. Simon (1885) described the new genus *Biantes* in the Phalangodidae, but compared it to *Hinzuanius* and *Stygnus*, both then in Gonyleptidae. *Biantes* originally included one species from India, and another from Madagascar. Thorell (1889), in a work on Burmese harvestmen, created the family Biantoidae to include only *Biantes* (known previously from India, Madagascar and from then on also Burma) and *Hinzuanius* (this one treated only briefly because it was outside the area of study, being an African genus). Soon afterwards, he (Thorell 1891b, Thorell 1891a) repeated the composition of Biantoidea restricted to *Biantes* alone. Sørensen (1896) confirmed the presence of the family in continental Africa,adding the new genus *Lacurbs* (from Cameroon), and including most of his EpedanoidaeSørensen 1886 (specifically, the Australasian *Ibalonius* and *Mesoceras*, plus the African *Hinzuanius*). Loman (1898) expanded the known distribution of *Biantes* in the Afrotropics, describing two new species from South Africa. Loman (1901) made a summary of the distribution of the family, mentioning the disjunctive areas in Africa (Cameroon, Abyssinia, many places in southern and eastern Africa and Madagascar) and SE Asia from Sri Lanka to Sumatra. Loman (1902) placed *Ibalonius* and *Mesoceras* elsewhere, created the new genus *Acudorsum* from the Seychelles and synonymized *Biantes* with *Hinzuanius*, thus recognizing only three genera *Acudorsum*, *Hinzuanius* and *Lacurbs* as Biantidae. Pocock (1902) followed the generic composition by Loman, but mistakenly thought that the family should take its name from the oldest genus, thus replacing Biantidae with Hinzuanidae. Roewer (1912) downgraded Biantidae to a subfamily of Phalangodidae, correctly using Biantinae instead of Hinzuaninae for the subfamily name. He revalidated *Biantes* from the synonymy of *Hinzuanius* and created the new genus *Heterolacurbs* from “Togo” as well as a few species in the other genera. From then on, during decades the Biantinae were gradually expanded with descriptions of new genera and species (*e.g*, Roewer 1923; Lawrence 1965; Martens 1978) without changes in the concept of the subfamily. Henriksen (1932) basically kept Roewer’s arrangement, only raising subfamilies and families one rank, so his Phalangodoidea included a family Biantidae, which was equivalent to Biantinae and not meant as a rupture with Roewer’s system. For many years the Biantidae were the same as today’s Biantinae, until Mello-Leitão (1938) included in Biantidae three of Roewer’s (Roewer 1923) subfamilies of Phalangodidae — the Biantinae, Stygnommatinae and Dibuninae. This proposal went universally ignored in the 1940s-60s, and those continued to be treated as subfamilies of Phalangodidae (*e.g*, Roewer 1949 for Stygnommatinae; Kauri 1961 for Biantinae). Lawrence (1959) created the new subfamily Lacurbsinae without detailed explanations (*i.e.* did not treat it in his paper, because it was only concerned with fauna of Madagascar). This subfamily went largely ignored until being resurrected by Kury (2003). Šilhavý (1973) was the first to observe Mello-Leitão’s proposal, recognizing for the first time in 60 years the Biantidae as a separated family, including the Biantinae, Stygnommatinae and Dibuninae (as in Mello-Leitão 1938) plus the new Caribbiantinae Šilhavý 1973for Caribbean species. Martens (1978) significantly treated the Biantidae as a separate family, solidifying the standard from then on. Also, following Šilhavý (1973), Suzuki (1977) and Kauri (1985) accepted Dibuninae as a biantid. Kury (2003) was the first (reaffirmed in Kury 2007) to assign Dibuninae to the Epedanidae, removing them from the Biantidae. Kauri (1985) added the monotypic subfamily Zairebiantinae from Central Africa. Pinto-da-Rocha (1995) discovered the Caribbiantinae also had representatives in South America, having been previously described by Roewer as Stenostygninae, hitherto regarded as a subfamily of Stygnidae (or Gonyleptidae), which had priority over Šilhavý’s family group name.

**Samoidae.** Samoidae was described in a paper where Sørensen (1886) created many other familial subdivisions in the Laniatores. It initially included *Badessa*, *Feretrius* and *Samoa*, all from Pacific islands. Roewer (1912) proposed a retrograde classification which endured for decades, recognizing a huge meaningless Phalangodidae including many of Sørensen’s families. So Samoidae became Phalangodidae Samoinae. Roewer also added to Samoinae the genus *Mitraceras* previously described from the Seychelles by Loman (1902) in Assamiidae. Roewer (1933) described the 5th genus of the subfamily, *Psyctrapus* from Costa Rica, as the first acknowledged Neotropical Samoinae. Roewer (1949) expanded considerably the Samoinae, with addition of *Microconomma* Roewer, 1915 (from Cameroon, originally placed in Phalangodinae) and the new genera *Badessania* (from the Australian continent), *Sawaiellus* (from Samoa), *Waigeucola* (from Indonesian Papua) and *Maracaynatum* (from Venezuela), the latter as the first South American Samoinae. Goodnight and Goodnight (1957) then described *Parasamoa* from Micronesia, while Lawrence (1959) described three new genera from Madagascar: *Anaceros*, *Hovanoceros* and *Malgaceros*, and Roewer (1963) added the Australasian *Fijicolana*. Šilhavý (1977) described two new genera – *Arganotus* and *Akdalima* – from Mexican caves (*Arganotus* known also from epigean milieu). Soon afterwards, Šilhavý (1979) described four new genera from the Caribbean (*Hummelinckiolus*, *Orsa*, *Reventula* and *Vlachiolus*), mentioning the other existing genera, but overlooking the ones from Madagascar. Goodnight and Goodnight (1983) transferred *Pellobunus* Banks, 1905 from Costa Rica to Samoinae, synonymizing *Psyctrapus* with it. Rambla (1984) studied material from the Seychelles, added a new *Mitraceras*, the first Seychellan *Samoa* and the new genus *Benoitinus* (with the first anophthalm species). Staręga (1989) placed *Microconomma* in an undescribed family, which only much later was formally described as Pyramidopidae (Sharma et al. 2011b). Critically, Staręga (1992) restored Samoidae as a family and considered it to be closest to Biantidae than to Phalangodidae. He also removed *Anaceros* to the Biantidae and transferred *Tetebius* Roewer, 1949 from the Phalangodidae to the Samoidae. *Microconomma* reappeared in his list of Samoidae without comment. Kury (2003) followed Staręga and considered Samoidae as a family. He also transferred to Samoidae *Cornigera* González-Sponga, 1987 and *Neocynortina* Goodnight & Goodnight, 1983 from Phalangodinae and *Kalominua* Sørensen, 1932 from Minuidae. Pérez-González and Kury (2007b) noted that typical samoids are restricted to Polynesia, Melanesia, Australia, Mexico, the West Indies, and Venezuela and cast doubt upon the samoid kinship of the Indonesian and African species. They also transferred the Australian *Zalmoxista* Roewer, 1949 from Phalangodinae to Samoidae. Currently this family is not subdivided into subfamilies.

**Stygnommatidae. Roewer (1923)** created Stygnommatinae as a subfamily of Phalangodidae, to include only the monotypic genus *Stygnomma* Roewer, 1912, previously in Phalangodinae. Later, Roewer (1927) added another monotypic genus, *Stygnomimus*, from the Riau Archipelago. Roewer (1928) erected the new genus *Stygnommatiplus* for two species previously described from Costa Rica and Panama and also *Zygobunus*, originally in Gonyleptidae, resulting in 4 genera. Roewer (1933) added a 5th genus, *Poascola*, from Costa Rica. Meanwhile, both attemps to elevate Stygnommatinae to family (Henriksen 1932 and Mello-Leitão 1949) were ignored. Goodnight & Goodnight added *Antagona* from Puerto Rico (Goodnight and Goodnight 1942) and *Flaccus* from Mexico (Goodnight and Goodnight 1947). Goodnight and Goodnight (1951) then perpetrated a big step backwards, by synonymizing all genera into *Stygnomma*, including that in Phalangodinae, and merging five different species under the name*Stygnomma fuhrmanni*. In the 1970s and 1980s, several new species of “*Stygnomma*” were described just adding diversity, but without any change in the composition of the group and keeping it in Phalangodinae (*e.g.* Rambla 1976; Soares and Avram 1981). González-Sponga (1987) finally restored Stygnommatinae as a subfamily of Phalangodidae, from the synonymy of Phalangodinae. In his Ph.D. thesis, Kury (1993) restored Stygnommatidae to family, although this change officialy took years to appear in press (Kury and Cokendolpher 2000). Pérez-González (2006) produced a Ph.D. thesis focusing on this family, and proposing many important changes, although haven't yet appeared in print through other articles.

### Incertae sedis

As part of the present project we made an enormous effort in order to solve the huge amount of taxa *incertae sedis* that are currently included in the Grassatores. A total of 32 taxonomic changes were proposed in the companion paper to this contribution (Kury and Pérez-González 2015), but a considerable amount of taxa still remain without any suprafamiliar allocation. A total of 59 genera and 81 species are not currently clearly assigned to any one grassatorean family. From this total, only the genus *Phalangodella* with seven species is considered as a Zalmoxoidea *incertae sedis*, while the rest remain for the moment simply as Grassatores *incertae sedis*. The major reason to retain that amount of taxa as uncertain is because we do not yet have feasible evidence to support either their familial or superfamilial assignment. The most common reason responsible for this picture is the absence of enough detailed, accurately illustrated descriptions for both sexes (*e.g.* omitted in Roewer 1923, Roewer 1949) and further need of detailed study of the male genitalia. In modern papers, the male genitalia became a mandatory set of characters to be included in taxonomical descriptions of Opiliones, and their importance for familial allocation have been continuously proved and reinforced (*e.g.* Pérez-González 2011; Pinto-da-Rocha et al. 2012, Kury 2014). But in some cases, even addition of the morphological information from the male genitalia is not enough to decide the familial placement (*e.g. Phalangodella* spp.). This is an indication that our current knowledge about Opiliones diversity still is unsatisfactory and evidence of this is the great amount of new families proposed in the last decades (*e.g.*Kury 2014, Kury and Villarreal M. 2015). New discoveries mainly arise from the study of the tiny, cryptic and litter-dwelling harvestmen fauna. Other familial groups of Opiliones likely still remain to be described or reconsidered, and an important aspect of this will be the study of the taxa here still considered as *incertae sedis*. Molecular works have been playing an important role in pointing out some research targets and systematic steering. Some interesting insights about several remaining *incertae sedis* have been pointed by the most recent molecular study of many lineages discussed here (*e.g.* Sharma and Giribet 2012) such as *Costabrimma* sp. nested inside *Icaleptes* spp. (therefore rendering *Icaleptes* as paraphyletic), *Turquinia cf. montana* recovered as part of Zalmoxoidea, as well as *Parascotolemon*, *Phalangodella* and *Urachiche* nested outside of Zalmoxidae. Undoubtedly the future points to the need for much further work to improve the systematics of Samooidea/Zalmoxoidea. We need to make the taxon sampling denser, explicitly include the name-bearing taxa, unveil the taxonomical identity of the terminals used in the previous molecular studies, and all of those together alongside detailed morphological descriptive work.We expect synergic action and feedback between molecular and morphological based research will increase our knowledge of harvestmen systematics in a near future, and help reduce the amount of remaining *incertae sedis* in the group.

## General description

### Purpose

 This project is a checklist of all valid specific and subspecific names (counted together) of the arachnid order Opiliones. Theproject intends to deliver 12 parts for ease of handling and preparing manuscripts. This is part 2 of 12, which covers the Grassatores *incertae sedis* as well as the two grassatorean superfamilies Samooidea and Zalmoxoidea.

## Project description

### Title

World Checklist of Opiliones species (Arachnida).

### Personnel

Adriano B. Kury (Author, Content Provider, Metadata Provider), Amanda C. Mendes (Author, Content Provider). Abel Pérez-González (Author, content provider). Daniele R. Souza (Author, Content Provider).

### Design description

This project aims to produce a general checklist of all the valid species and subspecies (which are countedtogether) names of harvestmen of the world (Arachnida, order Opiliones). That is, only senior homonyms and synonyms are included. Alternative unused combinations are not listed.

### Funding

This study has been supported by grants # 562149/2010-4 (PROTAX- OPESC project), # 504327/2012-7 (Sistema deInformacões sobre a Biodiversidade Brasileira (SiB-Br) - Coleções Biológicas) and scholarship # 302116/2010-9 (PQ - AMMA project) from the Conselho Nacional de Desenvolvimento Cientffico e Tecnológico (CNPq) to ABK/DRS and grant FONCyT PICT 2011-1007 to APG.

## Geographic coverage

### Description

General spatial coverage: worldwide. There is no Laurasian (Nearctic + Palearctic) representative of the groups treated here. Both Samooidea and Zalmoxoidea reach their diversity peak in the Neotropics.

The 2 superfamilies plus the unassigned taxa comprise 341 Neotropical species, 91 Afrotropical, 62 Australasian, 62 Indomalayan, totaling 556 species worldwide.

## Taxonomic coverage

### Description

**Figure 1.**
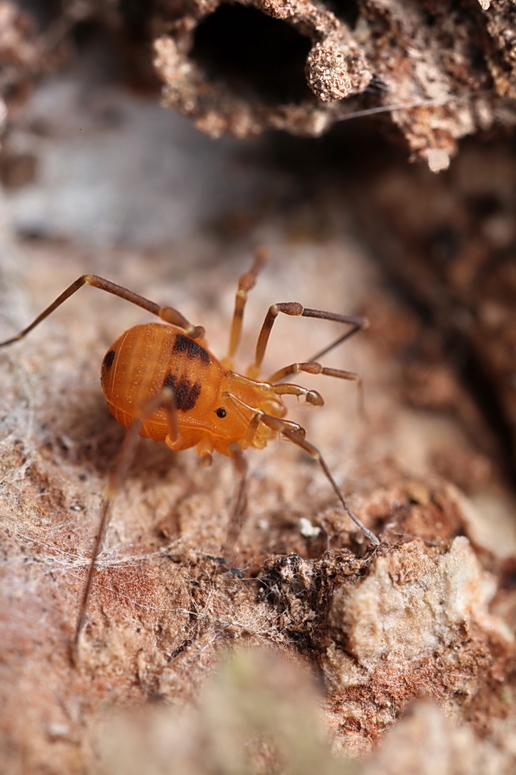
Biantidae, *Biantes* sp., from Singapore. Photo courtesy James Koh.

The Samooidea include 3 families:

Biantidae Thorell, 1889 (Fig. 1), (= Hinzuanidae Pocock, 1903), with 131 species from the tropics, absent from Laurasia.

**Figure 2.**
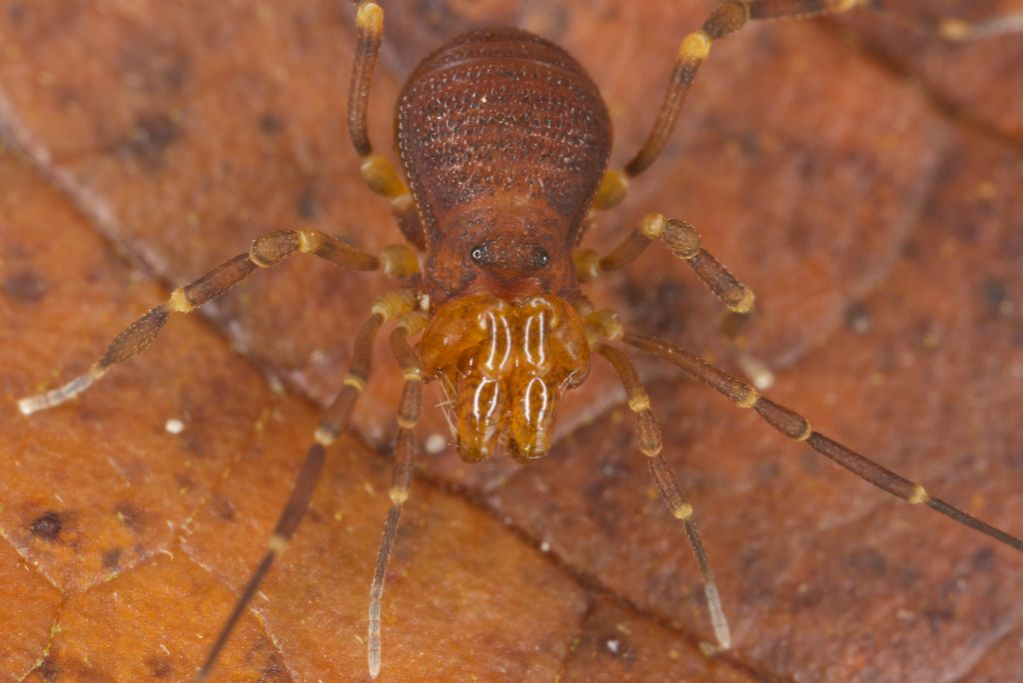
Samoidae, Pellobunus cf. insularis Banks, 1905, from Boca del Toro, Panama. Photo courtesy Gonzalo Giribet.

Samoidae Sørensen, 1886 (Fig. 2), with 48 species, mainly from Neotropics, but also from Australasia and Seychelles.

**Figure 3.**
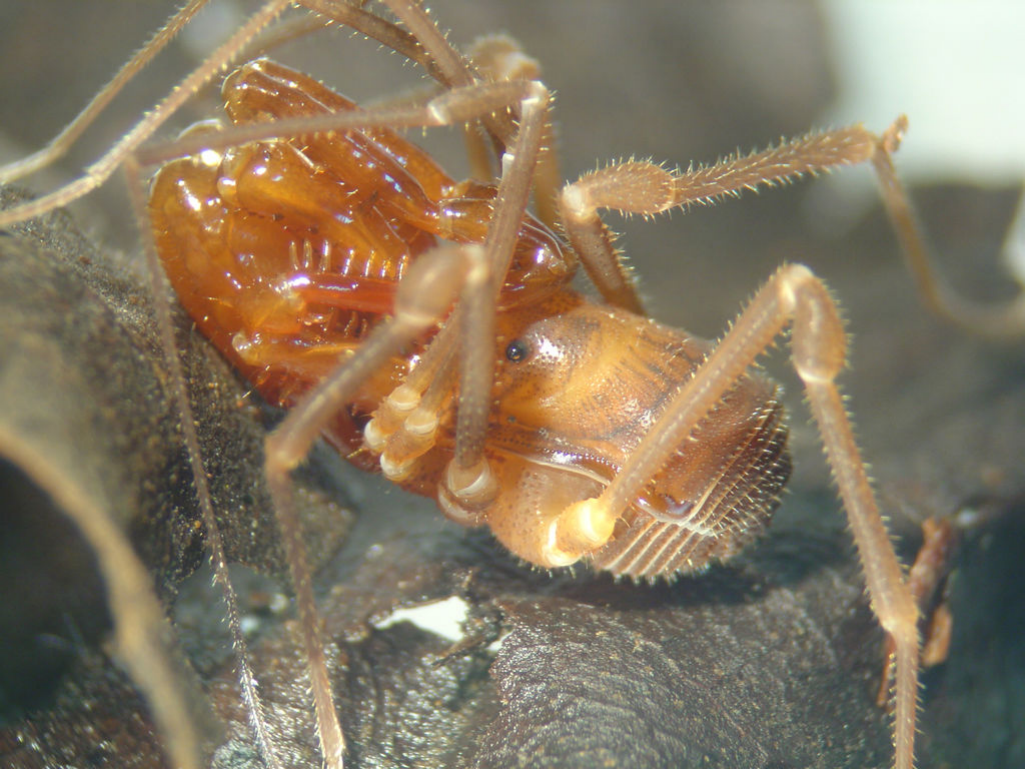
Stygnommatidae, *Stygnomma* sp., from Siquirres, Costa Rica. Photo by Abel Pérez-González.

Stygnommatidae Roewer, 1923 (Fig. 3), with 33 species, all Neotropical.

**Figure 4.**
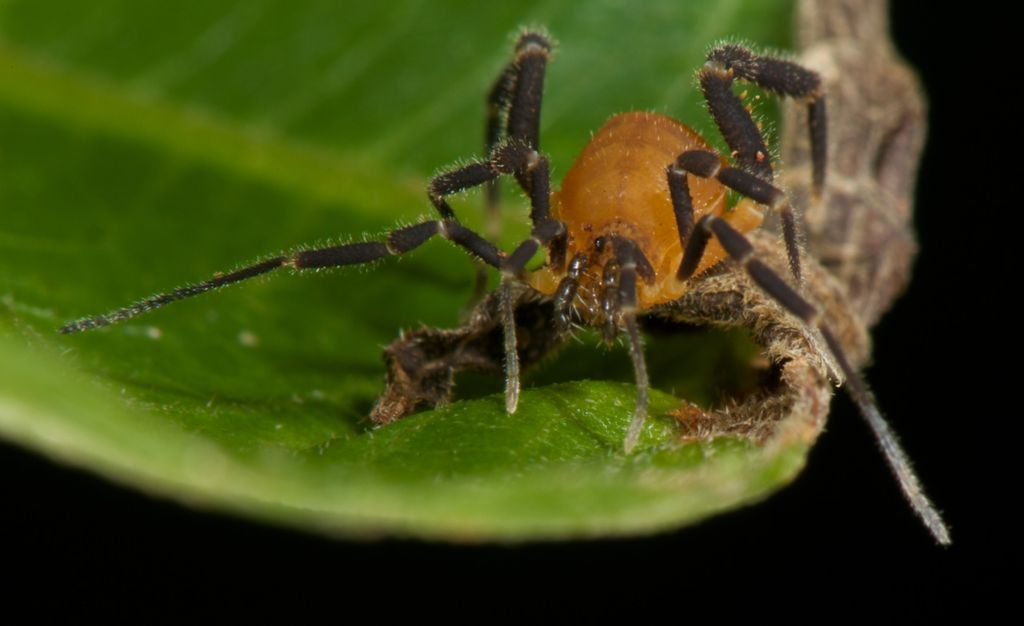
Zalmoxidae, *Pirassunungoleptes* sp., from Amazonas, Brazil. Photo and ID courtesy Gonzalo Giribet.

**Figure 5.**
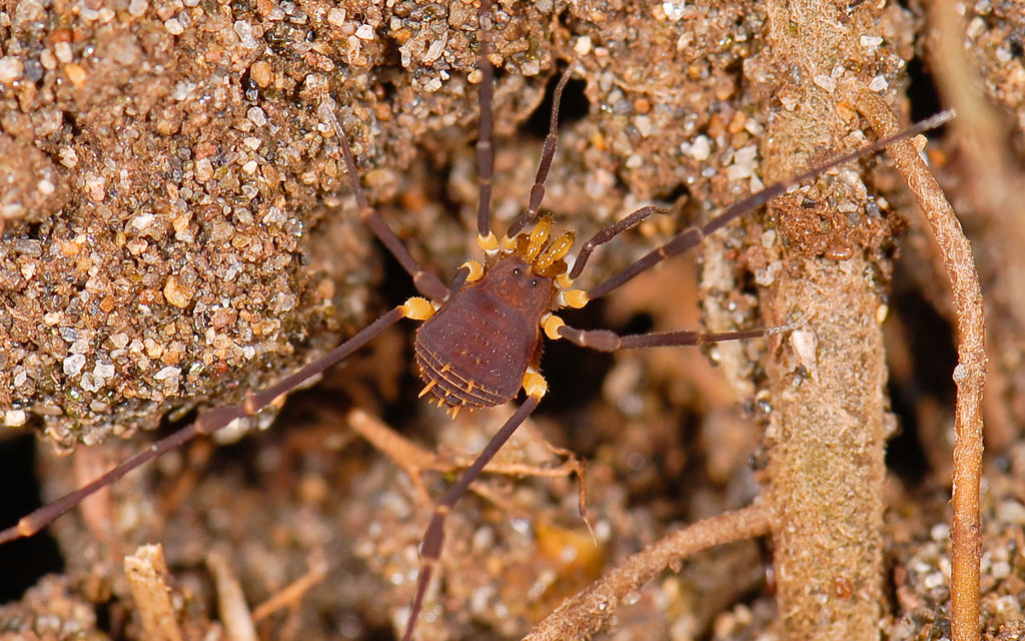
Zalmoxidae, *Panopiliops reimoseri* (Roewer, 1949), from Botanical Garden, Laguna Lodge, vic. Tortuguero, Costa Rica. Photo and ID courtesy Marshal Hedin. Picture online at link.

The Zalmoxoidae include 6 families - Fissiphalliidae Martens, 1988, Escadabiidae Kury & Pérez, 2003, Kimulidae Pérez-González et al. 2007 (= Minuidae Sørensen, 1932, unavailable name), Guasiniidae González-Sponga, 1997, Icaleptidae Kury & Pérez, 2002 and Zalmoxidae Sørensen, 1886 (= Minuidinae Mello-Leitão, 1933, = Stygnoleptinae H. Soares, 1972) (Fig. 4, Fig. 5). Of these, 4 are microdiverse, whilst the larger Kimulidae has 30 species and only Zalmoxidae has 219 species.

### Taxa included

**Table:** 

Rank	Scientific Name	Common Name
kingdom	Animalia	animals
phylum	Arthropoda	arthropods
class	Arachnida	arachnids
order	Opiliones	harvestmen
suborder	Laniatores	
infraorder	Grassatores	
superfamily	Samooidea	
family	Biantidae	
family	Samoidae	
family	Stygnommatidae	
superfamily	Zalmoxoidea	
family	Escadabiidae	
family	Fissiphalliidae	
family	Guasiniidae	
family	Icaleptidae	
family	Kimulidae	
family	Zalmoxidae	

## Temporal coverage

**Living time period:** Recent.

## Usage rights

### Use license

Open Data Commons Attribution License

## Data resources

### Data package title

KURY, A.B. & SOUZA, D.R. (2014) Part 2: Laniatores – Samooidea, Zalmoxoidea and Grassatores incertae sedis. Opiliones2

### Resource link

GBIF: http://ipt.pensoft.net/ipt/resource.do?r=opiliones2

### Number of data sets

1

### Data set 1.

#### Data set name

Darwin Core Archive World Checklist of Opiliones species (Arachnida). Part 2: Laniatores – Samooidea, Zalmoxoidea and Grassatores incertae sedis

#### Data format

Darwin Core Archive format

#### Number of columns

20

#### Character set

UTF-8

#### Download URL

http://ipt.pensoft.net/ipt/archive.do?r=opiliones2

#### Data format version

1.0

#### Description

**Data set 1.** 

Column label	Column description
taxonID	sequencial number
type	Checklist
basisOfRecord	Dataset
kingdom	Animalia
phylum	Arthropoda
class	Arachnida
order	Opiliones
suborder	Laniatores
superfamily	in this part it is either Samooidea or Zalmoxoidea or none.
family	quite few identified families, blank for incertae sedis at this level.
subfamily	blank for families not subdivided in subfamilies.
genus	a single word starting in Upper case
specificEpithet	a single word starting in Lower case
infraspecificEpithet	a single word starting in Lower case. But it is very rare in this set. Mostly it is blank.
scientificNameAuthorship	author and year, separated buy a comma, sometimes in parentheses.
scientificName	concatenation of the previous 4 columns.
taxonRank	species or subspecies
realm	each of the 6 WWF biogeographical realms
taxonomicStatus	they are all valid at this stage. In the future the database may also contain junior synonyms and replaced homonyms
rightsHolder	Kury, Adriano B. -- the coordinator of the entire project

## Supplementary Material

Supplementary material 1 World Checklist of Opiliones species (Arachnida). Part 2: Laniatores – Samooidea, Zalmoxoidea and Grassatores incertae sedis Data type: occurences
Brief description: This database is part 2 of 12 of a project containing an intended worldwide checklist of species and subspecies of Opiliones, and it includes the superfamilies Samooidea and Zalmoxoidea plus the Grassatores currently not allocated to any family. In this Part 2, a total of 556 species and subspecies are listed
File: oo_32914.xlsx AB Kury & DR Souza
